# Optimization and control of the light environment for greenhouse crop production

**DOI:** 10.1038/s41598-019-44980-z

**Published:** 2019-06-17

**Authors:** Pingping Xin, Bin Li, Haihui Zhang, Jin Hu

**Affiliations:** 1 College of Mechanical and Electronic Engineering, Northwest A&F University, Yangling, Shaanxi 712100 China; 2 Key Laboratory of Agricultural Internet of Things, Ministry of Agriculture and Rural Affairs, Yangling, Shaanxi 712100 China; 3 Shaanxi Key Laboratory of Agricultural Information Perception and Intelligent Service, Yangling, Shaanxi China

**Keywords:** Computational models, Light responses

## Abstract

Optimization and control of the greenhouse light environment is key to increasing crop yield and quality. However, the light saturation point impacts the efficient use of light. Therefore, the dynamic acquisition of the light saturation point that is influenced by changes in temperature and CO_2_ concentration is an important challenge for the development of greenhouse light environment control system. In view of this challenge, this paper describes a light environment optimization and control model based on a crop growth model for predicting cucumber photosynthesis. The photosynthetic rate values for different photosynthetic photon flux densities (PPFD), CO_2_ concentration, and temperature conditions provided to cucumber seedlings were obtained by using an LI-6400XT portable photosynthesis system during multi-factorial experiments. Based on the measured data, photosynthetic rate predictions were determined. Next, a support vector machine(SVM) photosynthetic rate prediction model was used to obtain the light response curve under other temperatures and CO_2_ conditions. The light saturation point was used to establish the light environment optimization and control model and to perform model validation. The slope of the fitting straight line comparing the measured and predicted light saturation point was 0.99, the intercept was 23.46 and the coefficient of determination was 0.98. The light control model was able to perform dynamic acquisition of the light saturation point and provide a theoretical basis for the efficient and accurate control of the greenhouse light environment.

## Introduction

Light provides energy for photosynthesis, and it is one of the most important conditions affecting the growth and development of the external environment crops^1^. It is difficult to meet light demands of crops in greenhouse due to the influence of covering materials, sun height angles and dip angle structures^2,3^. The greenhouse light usually appears to be weak at certain times of the year, especially in winter, early spring, and rainy seasons. This weak light situation inhibits the growth of the stem diameter and reduces dry matter accumulation, the net photosynthetic rate decreases due to nonstomatal limitation, the carboxylation efficiency and maximum RuBP regeneration rates also decrease^4,5^; These phenomena slow down the growth and development of crops and make them vulnerable to pests, causing more fallen leaves, less flowers and fruits^6–8^. The yield and quality of crops are seriously affected. Therefore, light environment control in greenhouse has been widely studied. Liu *et al*. considered the effect of the light quality on crop growth and designed an adjustable LED system capable of controlling light quality. The authors verified the effect of this system on plant growth and development through a spinach growth experiment^9^. The control method initially considered crop demands, but there were shortcomings in the dynamic regulation mechanism. Recently, related research has been performed on dynamic light environment regulation. Zhang *et al*. studied how to accomplish greenhouse light filling accurately by adjusting light quality^10^. Pinho *et al*. studied the dynamic control of supplemental light in greenhouse environment^11^. The above studies used a fixed control target value method to achieve dynamic feedback control of light quality and intensity through the real-time monitoring of temperature, photosynthetic photon flux densities (PPFD), and light quality. These studies laid a good foundation for environmental light control researches. However, these studies did not consider the internal correlation between the light saturation point and PPFD, CO_2_ concentration, temperature, and other environmental factors affecting crop growth^12–14^. These factors exhibit strong dynamic change over time^15^, which leads to significant changes in the photosynthetic rate and light saturation point. Therefore, to improve the photosynthetic rate, the efficient control of the greenhouse light environment needs to be done. Specifically, an optimal control model of the light environment need to be built, which considers the influence of the temperature, PPFD, and CO_2_ concentration on photosynthetic rate.

An photosynthetic optimization and control model should be established based on the light saturation point^16^. In this context, scholars have performed a large amount of researches on light saturation point acquisition^17–21^. Based on measuring light response curves, the software package Photosyn Assistant, photosynthesis workbenchand and statistical regression methods^22–25^ are used for light response curve fitting and the light saturation point acquisition. The common statistical regression methods are rectangular hyperbola model, non-rectangular hyperbola model, index model and modified rectangular hyperbola model. The differences in crop light response characteristics under different physiological conditions lead to tremendous differences in the light saturation points extracted by different models and methods^26,27^. Among these methods, the predicted light saturation point obtained by the modified rectangular hyperbola model has a high accuracy. With light increases, light response curve first increases rapidly then enters a flat region in which the photosynthetic rate increases slowly then decreases^28^. However, the peak value of the flat region, which is light saturation point, obtained by existing experimental methods and regression fitting is not accurate enough. The generalizability and accuracy of the modified rectangular model need to be improved when dealing with different light response curves. Therefore, studying the optimization method of multiple function flat areas in the crop light environment regulation system to achieve precise and fast acquisition of the light saturation point under different conditions has become the key scientific problem involved in. To achieve precise and fast acquisition of the light saturation point under different conditions, the optimization method for maximum value in flat region needs to be studied. This method is the key to build a high-precision light environment dynamic control model.

Recently, to solve the above problems, intelligent algorithms designed for target value optimization have been widely used in solving dynamic optimization problems^29^. Among these algorithms, the genetic algorithm (GA) is the earliest optimization method. The GA has rapid global search ability and fast convergence speed, and has been widely used in agriculture^30,31^. Zhang *et al*. studied an optimal irrigation model for winter wheat yields based on the GA^32^. Wu *et al*. studied the optimal layout of soil moisture sensors based on the GA^33^. However, they did not use feedback information in the system, which lead to the poor local searching ability. To improve the optimization accuracy, a new swarm intelligence optimization algorithm named ant colony optimization (ACO) algorithm was proposed^34–36^. This method offers very strong robustness and the ability to search for better solutions. Therefore, it has spread widely and achieved good results^37^. The above researches provide a theoretical basis for light saturation point dynamic optimization. However, they used different parameters, optimization conditions and functions in algorithm designs, which causes difficulties in setting those options to design an algorithm with the best performance.

The objectives of this study were as follow: (1) establish photosynthetic rate prediction model based on the support vector machine (SVM), radial basis function (RBF) and back propagation (BP) algorithms, the prediction model with the best performance is used as the optimization objective function to obtain the light saturation point; (2) predict the light saturation point under different temperatures and CO_2_ concentrations using the ACO algorithm and GA, then compare the predicted light saturation point and measured light saturation point; (3) establish a light environment optimal control model, with temperature and CO_2_ as the input, light saturation point as the output, and provide a theoretical basis for the precise control of a greenhouse light environment.

## Materials and Methods

This experiment was performed in the glass greenhouse at Northwest A&F University from February to April 2016. The experimental plant is cucumber, and the variety is “Changchun Mici”. Plump cucumber seeds were selected, then swelling, germination, and low-temperature breeding were performed. Seedling operations were performed in the nutrition pots (540 mm × 280 mm × 50 mm × 50 holes). Agricultural special substrates with the same nutrient content were used in the nutrition pots. Parameters of the substrates were as follow: 50% (mass ratio) organic fertilizer, 20% (mass ratio) humic acid, PH values was 5.5–6.5. During the cultivation period, water and light were applied uniformly. Sowing lasted for 22 days. When the second true leaves of the cucumber seedlings were flattened, the cucumber seedlings with uniform growth were selected for the experiment. Normal field management was performed during the experiment.

**Figure 1:**
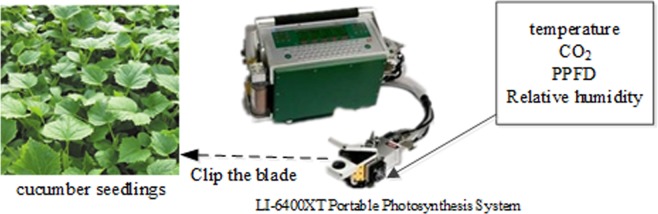
Schematic diagram of the test platform.

At noon, the temperature in the greenhouse was too high, and stomatal closure occurred in the cucumber plants, resulting in the “midday depression” phenomenon. Therefore, the plant parameters were measured and obtained over two periods: from 9:00 to 11:30 and from 14:30 to 17:30. During the experiment, the actual humidity in the greenhouse was 55–70%. To set the experimental conditions (environmental parameters) required for measurement, different sub-modules of a LI-6400XT Portable Photosynthesis System were used, as shown in Fig. 1. The CO_2_ selection injection module set 7 CO_2_ concentration gradients (600, 800, 1000, 1200, 1500, 1700, 2000 μmol/mol). The temperature control module set 6 temperature gradients (12, 15, 20, 25, 30, 33 °C). The LED light source module (6400-02B) spectral output has one red peak centered at about 670 nm and a blue peak centered at about 465 nm. The red: blue color ratio was set to be 9:1. The LED light source module set 10 PPFD gradients (100, 200, 300, 400, 500, 600, 800, 1000, 1200, 1400 μmol/m^−2^ s^−1^). Four hundred and twenty groups of experiments were completed over the above periods, and the average values for each plant were measured 3 times. The four hundred and twenty (420 =7 × 6 × 10) groups of experiment were obtained by nesting a combination of 7 CO_2_ concentration gradients, 6 temperature gradients and 10 PPFD gradients. Finally, four hundred and twenty sets of data samples were collected.

### Data analysis methods

As a representative output of plant growth, plant photosynthetic rate has a significant and nonlinear relationship with PPFD, temperature, and CO_2_ concentration. Artificial neural networks can discover this relationship by learning a large number of input and output samples. Therefore, the greenhouse light environment control model building process consists of three parts: building photosynthetic rate prediction model, obtaining light saturation point by photosynthetic rate optimization, and the optimization of the light environment control model. First, the 420 sets of data were normalized and entered into three types of algorithms (SVM algorithm, RBF algorithm, and BP algorithm) to train the model. A prediction model was then established based on the algorithm with the highest prediction accuracy. The photosynthetic rate prediction model network was used as the input of optimization objective function. Under the temperature and CO_2_ nested conditions, the photosynthetic rate prediction model network was instantiated to obtain the corresponding light response curves. The ACO algorithm and the GA algorithm were then used to obtain the light saturation points based on the light response curves. To obtain an optimal photosynthetic rate and the corresponding light saturation point, the light saturation points predicted by two algorithms were compared with the measured saturation point. The algorithm yielding the optimal light saturation point was chosen to build the light optimization and control model. Based on the discrete optimization results mentioned above, a nonlinear regression method was used to build the light environment control model, whose dependent variable was light saturation point and independent variables were temperature and CO_2_ concentration. At last, this model was verified.

### Construction of photosynthetic rate prediction model

**Figure 2:**
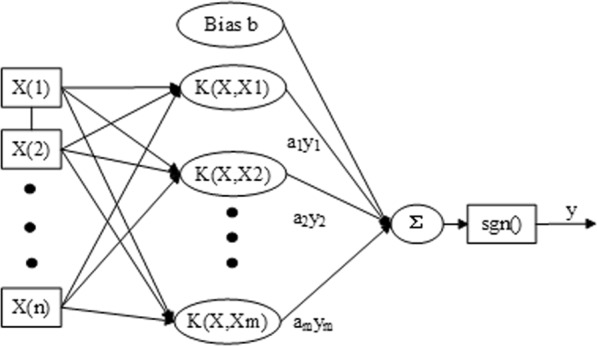
Support vector machine algorithm structure diagram.

Similar to the multilayer perceptron network and the radial basis function network, the SVM is a machine learning method that uses statistical theory to perform nonlinear classification and regression^38^. It intends to create a classification hyperplane as the decision surface and it finds the optimal classification plane which minimizes the distances between all training samples and the optimal classification plane. A schematic diagram of the SVM algorithm is shown in Fig. 2. Generally, SVM has the following advantages: strong generality, good robustness, the ability to construct functions over a wide variety of function sets, high effectiveness, no need for fine-tuning, and simple calculation. It is theoretically ideal, and is one of the best ways to solve practical problems. Therefore, SVM provides a feasible and effective way to predict the photosynthetic rate.

**Figure 3:**
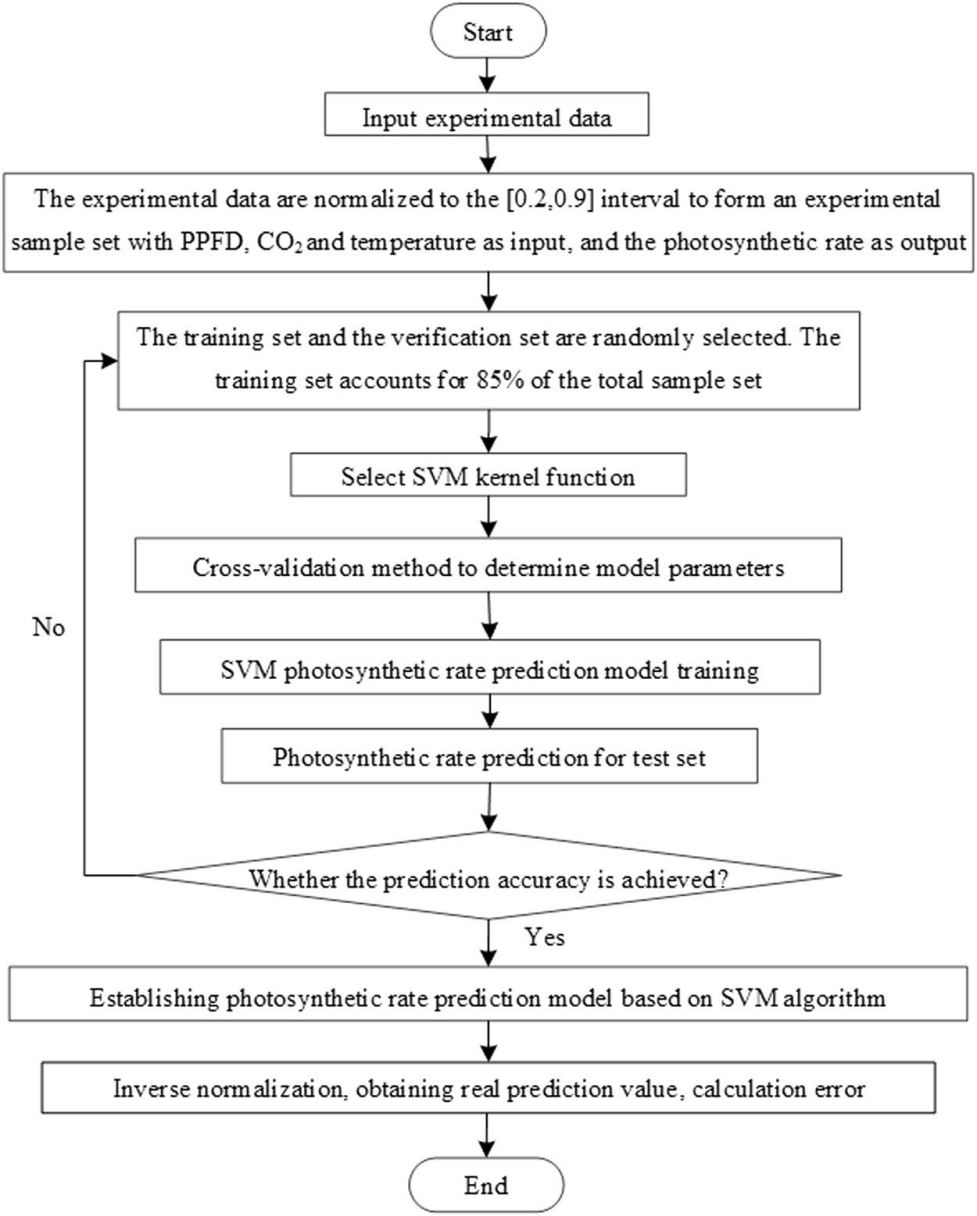
Flow chart of support vector machine algorithm.

For SVM, K is the kernel function shown in Fig. 2. This paper adopts the radial basis kernel function. The hidden node is the inner product of the input sample and a support vector, and the output node is the linear combination of the hidden layer output. The input variables of the photosynthetic rate prediction model are temperature, CO_2_ concentration and PPFD. The output target value is the photosynthetic rate. The construction process mainly includes test data normalization, devision of training sample and test sample sets, selection of kernel function and parameters, model training. Finally, accurate prediction of the photosynthetic rate was achieved. The specific model construction process is shown in Fig. 3.

During the model construction, 420 groups of experimental sample data were normalized then entered into the model.The interval is [0.2, 0.9], and the normalized function is

$$y={x}_{\min }+(0.9-0.2)\times (x-{x}_{\min })/({x}_{\max }-{x}_{\min })$$

A training sample set and a test sample set were randomly generated. 85% of the sample data were selected as the training set, and the remaining 15% of the sample data were collected as the validation set. The kernel function transformed the sample data in the low-dimensional nonlinear space into a high-dimensional eigenspace for linear regression. The regression function is as follows:

$$f({\boldsymbol{x}})=\sum _{i=1}^{nsv}(\overline{{\alpha }_{i}}-{\overline{{\alpha }_{i}}}^{\ast })K({{\boldsymbol{x}}}_{{\boldsymbol{i}}},{\boldsymbol{x}})+{\boldsymbol{b}}$$

where *K*(*x*_*i*_, *x*) is the kernel function, *x*_*i*_ is the support vector, nsv is the support vector number, and ***b*** is the bias. $\overline{{\alpha }_{i}},{\overline{{\alpha }_{i}}}^{\ast }$ is the Lagrange multiplier.

The SVM regression performance is primarily affected by the type of kernel function and model parameters^39^. The kernel function converts nonlinear inseparable samples into linear separable samples in the feature space. Different kernel functions make the SVM model produce different classification hyperplanes, which directly affects the model performance^40^. The radial basis function was selected in this study to construct the regression function. The function is expressed as follows:

$$K({{\boldsymbol{x}}}_{{\boldsymbol{i}}},{\boldsymbol{x}})=\exp (\,-\,{g}^{\ast }\,{\Vert {{\boldsymbol{x}}}_{{\boldsymbol{i}}}-{\boldsymbol{x}}\Vert }^{2})$$

The model’s primary influencing parameters are kernel parameter *g* and influence factor *c*, which affect the kernel function’s shape and the prediction accuracy, respectively. In this paper, the cross-validation method was used on different *c* and *g* combinations. And the optimal value for *c* was 2.82, and for *g* it was 0.50. Following the diagram illustrated in Fig. 3, the modeling process was repeated until it reached the predicted accuracy. Finally, the photosynthetic rate predicted value was obtained through reverse normalization.

This paper also used the BP algorithm^41^ and the RBF algorithm^42^ to construct photosynthetic rate prediction models, and the photosynthetic rate prediction and comparison analysis of the same verification set were carried out. The indicators selected for comparison were MAE, R^2^, MRE and RMSE^43^.

During the process, the BP algorithm adopted a three-layer network structure with 3 input layer neurons, 5 hidden layer neurons, and 1 output layer neuron. The neuron transfer function of hidden layer was S-type tangent function (tansig), the output layer transfer function was linear function, and the LM training method is used to train the BP network. In this study, the network training target error was 1.00 × 10^−4^, and the number of training steps was set to 1000. The RBF algorithm also adopted a three-layer network structure with 3 input layer neurons and 1 output layer neuron. The transformation of the input layer to the hidden layer was realized by a gaussian radial basis function, the output layer function was linear function, the expansion coefficient was 0.30, and the target error of network training was 1.00 × 10^−4^.

### Light saturation point optimization based on ACO algorithm

**Figure 4:**
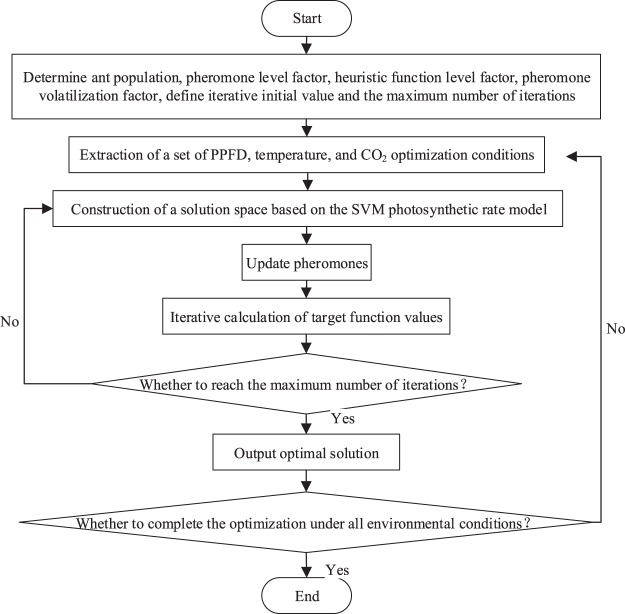
Flow chart of ant colony optimization algorithm.

As the SVM photosynthetic rate prediction model indicated, the photosynthetic rate and cucumber light saturation point varied significantly under different temperatures and CO_2_ concentrations. Therefore, to establish a precise and efficient light control model, maximum photosynthetic rates and corresponding light saturation points under different environmental parameters must was required. In this paper, the optimization algorithm consisted of two parts: the under nested conditions and the ACO algorithm. To build the target value optimization function, we instantiated the SVM photosynthetic rate prediction model and obtained light response curves under nested temperature and CO_2_ concentration conditions. The temperature gradient was set to 12–36 (°C), step size to 3 (°C). The CO_2_ concentration gradient was set to 300–2000 (µmol/mol), step size to 200(µmol/mol). The objective function used in this study was y (i) = svmfun (x(i),T,CO), where T is temperature, CO is carbon dioxide concentration, x (i) is PPFD, and y(i) is the photosynthetic rate. The ACO algorithm received and optimized the target value optimization function. Then, it constructed the solution space, updated the pheromone concentration, and iteratively searched for the optimal solution, which was the light saturation point value^44,45^. The flow chart of this process is shown in Fig. 4.

The ACO algorithm was initially applied to the discrete path optimization problem. The walking path of a single ant is used as a feasible solution to the optimization problem. The walking path of the entire ant population constitutes a solution space. Ants with shorter paths release more pheromones so that more ants can perceive and select the path. Finally, most of the ants gather in the optimal path with the highest pheromone concentration, which is the optimal solution to the problem.

The key to optimize the light response curve under specific temperature and CO_2_ concentration based on the ant colony algorithm is to transform the discrete space optimization problem into a continuous function optimization problem. Therefore, the discrete ACO algorithm was improved in three aspects: solution space construction, the ant colony transfer strategy, and the pheromone distribution function. The optimization method used in this study was as follows.

For solution space construction, the defined domain interval was set to [800, 1800] based on the PPFD distribution, the ant colony population was N = 50, and the length of each subsection was D = 20. Each ant i was placed in each middle area. The initial distribution of the ant colony is

$${x}_{i}=800+(\frac{i}{N}-\frac{1}{2})\times D$$

when the ant colony moves Δ*x*, the ant number changes to:

$${\rm{\Delta }}n=\frac{{\rm{\Delta }}x}{D}$$

As for the pheromone distribution function, the current ant colony information distribution is calculated based on the ant colony solution space coordinates, and the pheromone distribution function is defined as the light response curve at a specific temperature and CO_2_ concentration. The optimization target function is as follow:

$$f({x}_{i})=F({x}_{i},T,C){|}_{T={T}_{0},C={C}_{0}}$$

where *F* is the photosynthetic rate model, *T*_0_ is the current temperature and *C*_0_ is the current CO_2_ concentration.

The pheromone distribution function *f*(*x*_*i*_), the heuristic function *η*, and the pheromone volatilization factor are used to calculate the total amount of each subinterval pheromone:

$${\tau }_{i}=(1-\alpha )\times ({\int }_{iD}^{(i+1)D}f({x}_{i})dx+{\eta }_{i}{{\tau }_{{\rm{i}}}}^{\text{'}})$$

This function is expressed as the pheromone concentration in each subrange, and the last generation of the residual pheromone concentration is weighted, *τ*_*i*_ is the residual concentration of the previous generation of pheromones a, α = 0.1.

The number of ant colonies in each subrange is:

$${N}_{{\rm{i}}}=\frac{{\tau }_{i}}{\sum _{i=1}^{n}{\tau }_{i}}$$

Second, according to the exact ant colony number of each subinterval and last generation, the movement direction of the ant colony is determined, and each single ant coordinate value is updated after one movement. The above steps are repeated until the maximum number of iterations is reached, and the optimal ant colony coordinates values *x*_*best*_ and *f*(*x*_*best*_) are the outputs, which represent the light saturation point value and the maximum photosynthesis rate. The maximum number of iterations performed in this study was 200 generations.

To further evaluate the results of ACO algorithm, the GA algorithm was chosen for comparison. To optimize the maximum photosynthetic rate with GA, the population was initialized. The individual algebra NIND was set to 40, the maximum genetic algebra was set to 200, the binary digit of the variable was set to 20, GGAP was set to 0.95, the cross PX probability was set to 0.7, and the mutation probability pm was set to 0.01. Then, the SVM photosynthetic rate prediction model network was instantiated to obtain light response curves under nested temperature and CO_2_ conditions. The objective function y(i) = svmfun (x(i),T,CO) was determined and encoded into binary, the result was the fitness objective function. According to the fitness, operations such as selection, crossover, and mutation were performed to update the population and obtain the optimal solution for the current generation. Similar to natural evolution, this process was repeated to iteratively update the population until the optimization was completed, which meant the maximum photosynthetic rate and light saturation point were under a certain temperature and CO_2_ condition were obtained. Then, another set of temperature and CO_2_ optimization condition was selected, the above process was repeated until all the the light saturation points were obtained.

The above optimization algorithm was evaluated by the following indexes: MAE, MRE, RMSE, R^2^ and running time. The algorithm with higher precision was used to construct the control model of greenhouse light environment.

### Establishment and verification of optimization and control model of greenhouse light environment

According to the acquired discrete light saturation points and their corresponding environmental conditions, the cucumber light optimization and control model was constructed by using nonlinear regression method. With temperature and CO_2_ concentration as input variables and light saturation point as output variable. This continuous model could dynamically acquire the light saturation point under any CO_2_ concentration and temperature.

To verify the accuracy of this model, the light saturation point of cucumber under a certain temperature and CO_2_ concentration was measured. With the certain environmental condition as input, the predicted light saturation point value and compared with the measured value. 35 samples in the same glass greenhouse, which were not part of the test sample, were randomly selected as the verification samples. The LI-6400XT Portable Photosynthesis System was used to create leaf chamber conditions. The air temperature was set by the temperature control module to 12, 15, 20, 25, and 30 °C. The CO_2_ concentration, controlled by a small CO_2_ cylinder, was set to 300, 600, 800, 1000, 1200, and 1500 μmol/mol. The cucumber light response curve was measured under the temperature and CO_2_ nested conditions. Because the temperature monitoring module had an accuracy of ±0.20 °C, a repeated detection method was adopted in the test. When the temperature was stable, the light saturation points under the 5 temperature gradients were measured 3 times and the average values were calculated. Thereby, 35 light response curves and the corresponding measured light saturation points were obtained. The above temperature and CO_2_ concentration gradients were entered into the model, and 35 predicted light saturation points were calculated. The correlation between the measured and the predicted light saturation points was analyzed by a linear fitting method.

## Results

### Prediction results of the SVM photosynthetic rate prediction model

**Figure 5:**
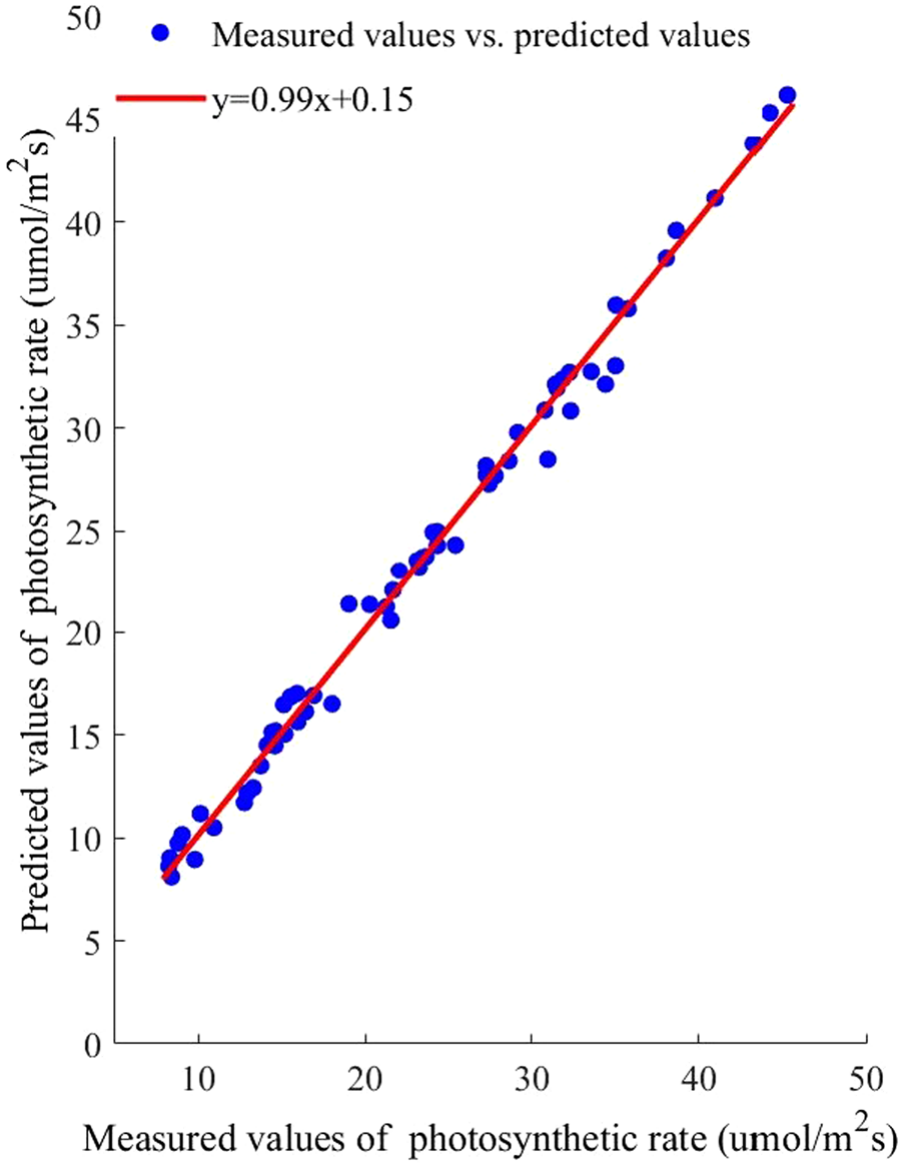
Correlation between measured photosynthetic rate values and predicted values based on support vector machine algorithm.

The experimental data were entered into the SVM algorithm, and an prediction model was built. The predicted photosynthetic rate values and the measured photosynthetic rate under the corresponding conditions were shown as scatter points in Fig. 5. The scatter points in the graph were fitted, and the fitting formula was $y=0.99x+0.15$. The slope of the fitting straight line was 0.99, and the intercept was 0.15, which indicated that correlation between the predicted and measured photosynthetic rate was high.

The determination coefficient (R^2^) of the fitting line was 0.99, indicating that the photosynthetic rate predicted value was highly correlated with the measured value. The root mean square error (RMSE) of the model was 0.95. In summary, the SVM photosynthetic rate model could accurately predict the photosynthetic rate under different environmental parameters and it provided a reliable optimization objective function for calculating the light saturation point.

### Prediction results of the RBF photosynthetic rate prediction model

**Figure 6:**
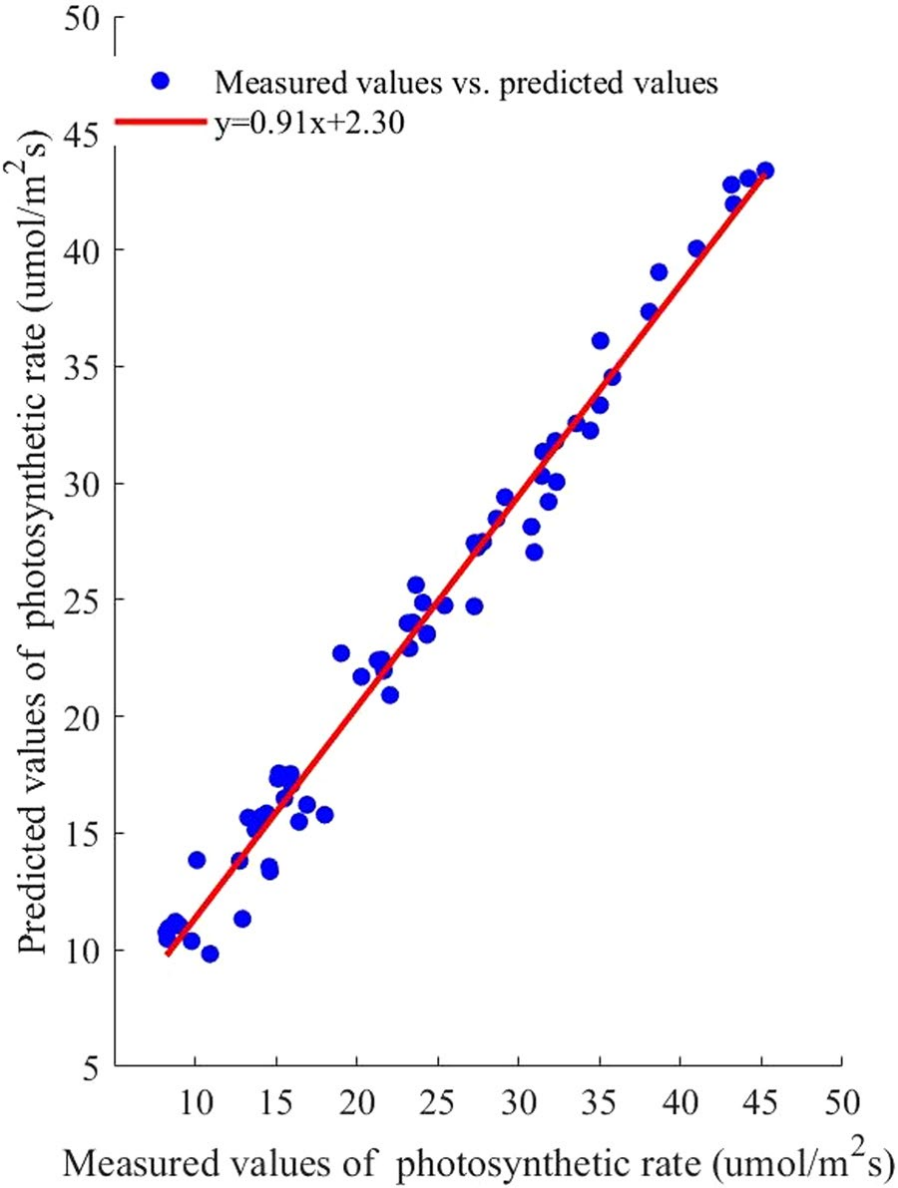
Correlation between measured photosynthetic rate values and predicted values based on radial basis function algorithm.

The experimental data were entered into the RBF algorithm, and the RBF photosynthetic rate prediction model was built. The predicted and measured photosynthetic rate values were shown as scattered points in Fig. 6. The R^2^ of the fitting line was 0.98 and RMSE was 1.36. The results indicated that the measured and predicted photosynthetic rates were highly correlated.

### Prediction results of the BP photosynthetic rate prediction model

**Figure 7:**
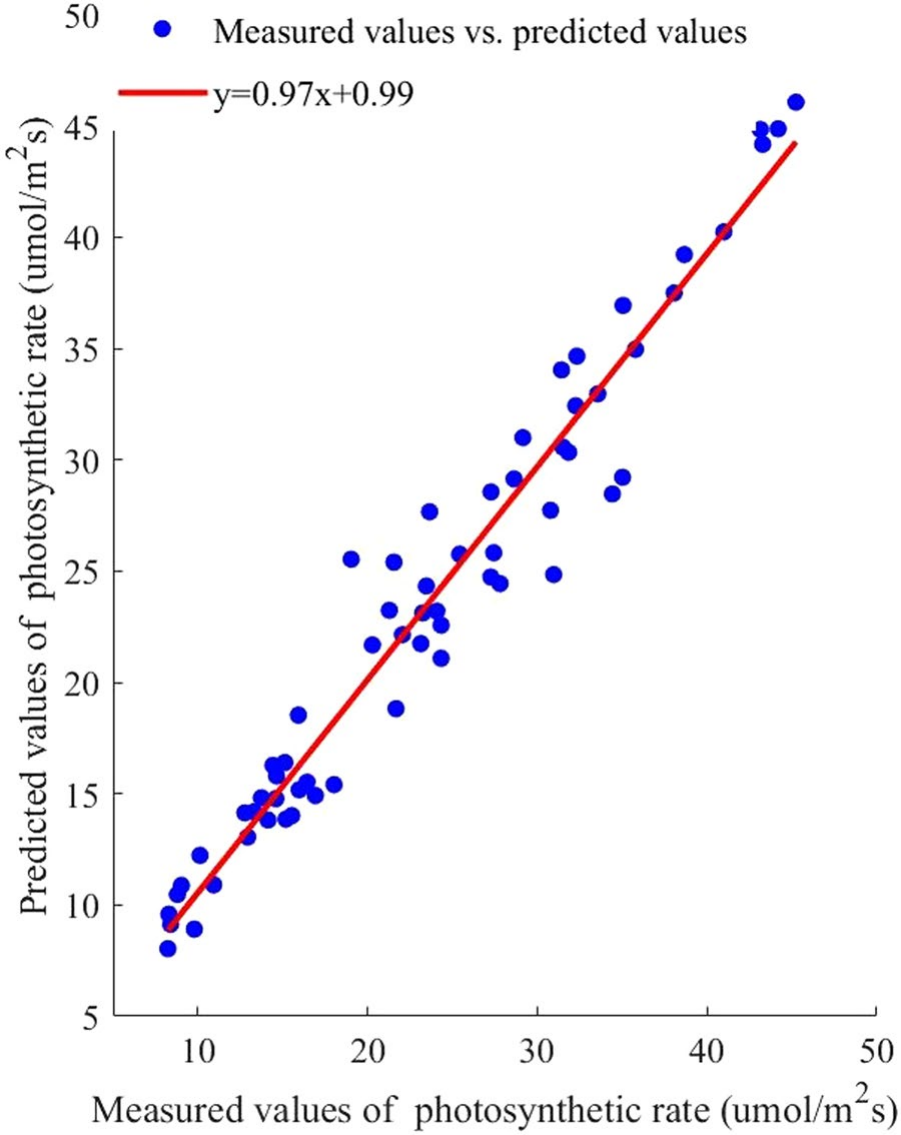
Correlation between measured photosynthetic rate values and predicted values based on back propagation algorithm.

The experimental data were entered into the BP algorithm, and a BP photosynthetic rate prediction model was built. The scatter points in Fig. 7 showed the predicted and the measured photosynthetic rate values. The R^2^ of the fitting line was 0.95 and the RMSE was 2.24,

### Comparison analysis for photosynthetic rate prediction model

To evaluate the performance of the above models, The mean absolute error (MAE), mean relative error (MRE), RMSE, R^2^ and running time were selected to analyze the prediction results. The result was shown in Table 1.

**Table 1:** The correlation between the measured photosynthetic rate and the photosynthetic rates predicted by the three algorithms.

prediction model	MAE	MRE/%	RMSE	R^2^	running time/s
SVM	0.86	0.89	0.94	0.99	12.32
RBF	1.18	2.99	1.36	0.98	89.11
BP	1.18	1.30	2.24	0.95	13.13

As shown in Table 1, the MAE of the SVM photosynthetic rate prediction model was the smallest. The MRE of the SVM photosynthetic rate prediction model was the smallest, indicating that this model offered the highest credibility. The RMSE of the SVM photosynthetic rate prediction model was the smallest, indicating that the SVM prediction model exhibited the highest accuracy. Also, the SVM prediction model had the highest R^2^. Thus, compared with other models, this prediction model was better at reflecting the essence of the sample data. Moreover, the SVM algorithm had the shortest running time, showed high computational efficiency. Based on the comparison and analysis of the above models, the SVM photosynthetic rate prediction model could predict the crops’ photosynthetic rate rapidly and accurately, which could provide a foundation for determining the light saturation point.

### Comparison of optimization results based on ACO algorithm and GA

Light response curves were obtained using the SVM photosynthetic rate prediction model network. The ACO algorithm and genetic algorithm(GA) were both used to optimize the maximum photosynthetic rate and calculate the light saturation point. The correlation between the measured light saturation points and the light saturation points predicted by the two algorithms was analyzed, and the evaluation indexes were MAE, MRE, RMSE, and running time. The correlation results obtained for the two algorithms are shown in Table 2.

**Table 2:** The correlation between the measured light saturation point and the light saturation points predicted by the two algorithms.

Optimization results	MAE	MRE/%	RMSE	running time/s
ACO	25.87	1.85	43.13	473.21
GA	75.38	5.33	102.08	1390.42

Table 2 showed that the MAE of ACO algorithm was 25.87, and MAE of GA was 75.38,which showed that the light saturation point predicted by the ACO algorithm was closer to the measured saturation point and could better reflect the crops’ real demand. The MAE of the ACO algorithm was only 1/5 of that of the GA. This discrepancy showed that the light saturation point obtained by the ACO algorithm could reflect the measured value better. Also, the RMSE of the ACO algorithm was smaller. At last, the running time of ACO algorithm was 1/3 of that of GA. Based on the above analysis, the ACO algorithm performed better, and could support the later modeling of light environment optimization and control target value.

### The greenhouse light environment optimization and control model construction

**Figure 8:**
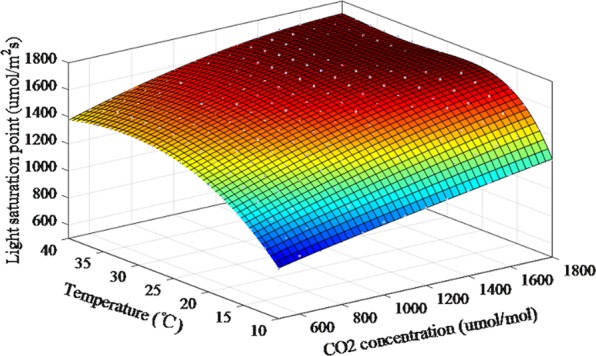
Light environment optimization regulation and control model.

Based on the optimization results of ACO algorithm, the light saturation points of each temperature and CO_2_ concentration gradient were obtained. The scatters were shown in Fig. 8. On this basis, A nonlinear fitting method was adopted to construct a multifactor coupling light environment optimization and control model for cucumber, where the temperature and CO_2_ concentration were independent variables, and the light saturation point was the dependent variable. The model formula was as follow:

1 $$\begin{array}{rcl}PPFD & = & f(T,C{O}_{2})=159.1-0.7938\times T+80.32\\  &  & \times C{O}_{2}+0.0002105\times {T}^{2}+0.162\times T\times C{O}_{2}\\  &  & -\,3.379\times C{{O}_{2}}^{2}-2.577\times {10}^{-5}\times {T}^{2}\times C{O}_{2}\\  &  & -0.00583\times T\times C{{O}_{2}}^{2}+0.09797\times C{{O}_{2}}^{3}\\  &  & +\,3.792\times {10}^{-7}\times {T}^{2}\times C{{O}_{2}}^{2}\\  &  & +\,6.997\times {10}^{-5}\times T\times C{{O}_{2}}^{3}-0.001279\times C{{O}_{2}}^{4}\end{array}$$

This three-dimensional model was shown in Fig. 8. The model’s R^2^ was 0.99, and RMSE was 7.79, indicating high fitting accuracy. The model could obtain a light saturation point under arbitrary temperature and CO_2_ concentration conditions and thus provide the basis for greenhouse light environment control.

### Verification of light environment control model

**Figure 9:**
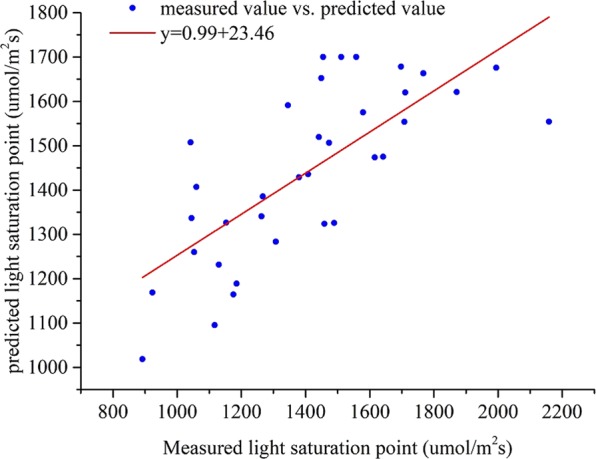
Correlation between measured light saturation point and predicted values based on ant colony optimization algorithm.

Regression fitting was performed to analyze the correlation between the measured and predicted light saturation points. The results were shown in Fig. 9. The slope of the fitting line was 0.99, the intercept was 23.46, and the R^2^ value was 0.98. These results indicated high fitness between the predicted and measured light saturation points and strong data reliability. Moreover, the optimization result was highly accurate.

## Discussion

Because the photosynthesis prediction models can only be established when the output variables are much fewer than the input variables, the SVM, BP, and RBF algorithms were used to build the photosynthesis prediction model, and their prediction performances were listed in Table 1. Given the same randomly selected training set, model built by SVM algorithm had the highest R^2^, the lowest RMSE, the lowest MAE, and the lowest MRE. This finding indicated that the predicted and measured photosynthetic rate was closely related, and the SVM algorithm not only exhibited the best calibration performance but also the best prediction performance. Furthermore, these results were consistent with those of Yong Wang and Linjie Duan^46,47^. As for process speed, the SVM model showed a shorter response time than the BP and RBF models, which illustrated that the SVM photosynthesis prediction model had the maximum operating efficiency. Thus, the SVM model could prevent some shortcomings of gradient-based learning methods.

The SVM photosynthesis prediction model exhibited better prediction performance than the BP or RBF model. The reasons are as follows. First, SVM algorithm is based on the structural risk minimization principle and has strong generalizability, which contributes to its good fitting effects and prevents it from overfitting. Second, SVM algorithm is used to solve convex optimization problems. Thus, the global optimal solution will definitely be found. However, BP algorithm uses a greedy learning strategy based on the search hypothesis space. In general, BP algorithm can only obtain a local optimal solution. Therefore, the SVM photosynthetic rate prediction model has the advantages of small storage space, strong algorithm robustness and high precision. Third, SVM algorithm applies the expansion theorem of kernel functions, a linear learning machine can be established in a high dimensional feature space without knowing the explicit function of nonlinear mapping. Therefore, compared with a linear model, the SVM algorithm avoids the “dimension disaster” problem while the computational complexity does not increase, but it also. Therefore, the SVM algorithm could construct the photosynthetic rate prediction model quickly and accurately.

To evaluate the optimization performance of ACO algorithm, the following parameters were given. MAE was 25.87, MRE was 1.85%, RMSE was 43.13 μmol/m^−2^ s^−1^, and running time was 473.21 seconds, indicating its better performance than GA. This result was consistent with other study^48^. The reasons for this finding are as follows. First, the ACO algorithm is a heuristic searching algorithm. It parameterizes the solution space, and the initial solution space has the same probability distribution. The probability distribution model is updated through pheromone accumulation, which guarantees that searches in the new model can be concentrated in the high-quality solution search space. Therefore, compared with the global searching method of GA, the evolutionary iteration speed of ACO algorithm is higher. It is easier for ACO algorithm to achieve the global optimal solution and obtain a more accurate photosynthesis rate and PPFD. Second, the ACO algorithm is a positive feedback algorithm. In the algorithm, the reason why the ants looking for food can find the best route is that the ant has accumulated a lot of information in the in their previous exploration. This pheromone accumulation produces a positive feedback process. Thus, the algorithm can obtain the optimal photosynthetic rate under different nesting conditions quickly and accurately. Finally, the ACO algorithm is a group parallel algorithm. Each ant has an independent searching operation for the same target. They begin to construct the solution space simultaneously at multiple points in the problem space. The solution to the whole problem will not be affected by the defects of individuals. Therefore, it was easy to obtain a higher optimization speed using the ACO algorithm to optimize the photosynthetic rate.

According to the optimization results of ACO algorithm, when the CO_2_ concentration was fixed, the cucumber’s light saturation points first rose rapidly with temperature increasing, then it slowed down, and finally it decreased. This trend was consistent with the law of crop growth^49^. The upward trend showed that not only did the maximum photosynthetic rate change with temperature, but the light saturation point did as well. This is because the activity of the enzyme in photosynthesis increased with the increase of the ambient temperature, thereby accelerating the photochemical reaction process. Above 30 °C, the light saturation point changed slowly as CO_2_ concentration increased. If the temperature continued to increase, the light saturation point would decrease. This was because the increase in temperature resulted in stomatal closure, which limited the plants’ photosynthetic capacity. When the temperature was constant, the light saturation point rose rapidly at first and then changed slowly with increasing CO_2_ concentration. This trend was observed because CO_2_ is an important substrate for photosynthesis^50^, and its concentration is one of the main factors limiting the rate of photosynthesis. Thus, there are different light saturation points at different CO_2_ concentrations. Related studies showed that the data in the cucumber fruiting period coincide with the trend of seedling data in this paper^51^. Therefore, according to crops’ light requirements under different light conditions, the establishment of a continuous cucumber seedling optimization and control model could improve crops’ photosynthetic rate, increase production and save costs.

In the existing researches, the quantitative light supplement mode is usually adopted to increase the light intensity by setting the thresholds and increasing the light duration. The results showed that the yield and quality of crops could be increased by supplementing light^52^. At the same time, cost analysis revealed that the cost of light could be recovered in one year, while the supplemental light could be used for many years^53^. Compared with the quantitative control mode, optimization regulation and control model can dynamically control the amount of supplemental light according to the PPFD requirement of crops in real-time environment, which can further reduce the waste of light energy and better meet the needs of crop growth for light.

## Conclusions

In this paper, the dynamic change of cucumber light saturation point with temperature and CO2 concentration was studied. Photosynthetic rate tests coupled with temperature, CO2 concentration and photon flux density were adopted during the cucumber seedling stage. The photosynthetic rate prediction models were established using the BP, RBF, and SVM algorithms. The three photosynthetic rate prediction models were verified and compared. The photosynthetic rate prediction model with the best performance was selected as the optimization target function. Based on this approach, the photosynthetic rate prediction model network was taken as the input, and CO_2_ concentration and temperature multigradient loop nesting was performed to instantiate the photosynthetic rate prediction model to obtain the light response curve. Moreover, the ACO and GA algorithm were used to optimize the maximum photosynthetic rate. The light saturation points were obtained, and the results were compared. The best algorithm results were used for the light optimization and control model construction. Based on the discrete optimization results, nonlinear fitting methods were adopted to construct the light environment optimization and control model, and the model was implemented for verification. The results showed that the model predicted the light saturation point accurately. The specific conclusions are as follows.

1. The factors affecting photosynthesis were sought based on the crop photosynthesis mechanism, there was a significant positive correlation between the PPFD, temperature and CO_2_ concentration and the photosynthetic rate. Under the temperature and CO_2_ concentration nested conditions, experiments were performed to obtain light response curves. The SVM, RBF, and BP algorithms were used to construct the photosynthetic rate prediction model to reflect the complex nonlinear relationship between the photosynthetic rate and the environmental factors. The results showed that the slope of the fitting line for the SVM photosynthetic rate prediction model was 0.99, the intercept was 0.15, the R^2^ was 0.99, the RMSE was 0.95 μmol/m^−2^s^−1^, the MAE was 0.86 and the running time was 12.32 seconds. The SVM photosynthetic rate prediction model exhibited high precision and generalizability compared with other models, providing theoretical supports for cucumber light environment control.
2. Based on the SVM photosynthetic rate prediction model constructed in this paper, light response curves under full-range temperatures and CO_2_ concentration conditions were obtained for instantiation. The ACO and GA algorithm were used to determine the maximum photosynthetic rate and then obtain the corresponding light saturation point. The results ACO algorithm showed that the MAE was 25.87, the MRE was 1.85%, the RMSE was 43.13 μmol/m^−2^s^−1^, and the running time was 473.21 seconds. Therefore, the light saturation point obtained by the ACO algorithm exhibited higher precision.
3. Based on the discrete results obtained by the ACO algorithm, a polynomial fitting method was used to establish the light environment control model. The R^2^ of the light optimization and control model was 0.99, the RMSE was 7.79. The predicted light saturation point was compared with the measured value to verify the accuracy of the light optimization and control model. The validation results showed that the slope of the fitted line between the measured and predicted values was 0.99, the intercept was 23.46, the R^2^ was 0.98, and the RMSE was 43.13 μmol/m^−2^s^−1^. The results showed that the light optimization and regulation model was highly accurate and could output the light saturation point under different temperature and CO_2_ concentration conditions, providing a theoretical basis for greenhouse light environment regulation. In addition, this method can be applied to establish crop control models in different greenhouses.
